# Knowledge, Attitudes, and Practices of Artificial Intelligence in Dentistry: A cross-sectional survey

**DOI:** 10.12688/f1000research.173028.2

**Published:** 2026-01-28

**Authors:** Usha GV, Bhuvaneshwari Nadar, Sultan Almalki, Tushar Bhagat, Inderjit M. Gowdar

**Affiliations:** 1 Public Health Dentistry, Bapuji Dental College and Hospital, Davangere, Karnataka, 577004, India; 2 Public Health Dentistry, Terna Dental College and Hospital, Nerul, Navi Mumbai, Maharashtra, 400706, India; 3 Preventive Dental Sciences, Prince Sattam bin Abdulaziz University College of Dentistry, Al Kharj, Riyadh Province, 11942, Saudi Arabia; 4 Prosthodontics, Prince Sattam bin Abdulaziz University College of Dentistry, Al Kharj, Riyadh Province, 11942, Saudi Arabia

**Keywords:** Dental students, Artificial Intelligence, Attitude, Knowledge, Practice

## Abstract

**Background:**

Artificial intelligence (AI) is rapidly reshaping various aspects of human life, including healthcare. In the Western world, AI is increasingly applied in education and clinical practice through algorithms designed to analyze health data, aid in prediction, and assist with disease diagnosis. However, developing countries like India face obstacles in adopting AI due to limited resources and socio-cultural factors.

**Aim:**

This study seeks to assess and compare the knowledge, attitudes, and practices related to AI in dentistry among undergraduate and postgraduate students in South India.

**Methodology:**

A descriptive cross-sectional online survey was conducted among dental students in South India. The survey included 21 validated, structured, close-ended questions addressing demographic details, self-assessment of knowledge, attitudes toward AI applications in dentistry, and self-perceived understanding of AI practice in the field.

**Results:**

Of 208 respondents (81.8% response rate), 95.6% were familiar with the term AI. Postgraduates demonstrated significantly greater awareness of AI applications (90.9%) compared to undergraduates (25.8%). About 78.3% of undergraduates believed AI supports diagnosis and treatment planning, while 33.4% of undergraduates and 43.2% of postgraduates expressed concern that AI may replace dentists in the future. Most respondents acknowledged AI’s role in oral radiology (UG: 79.1%; PG: 72.2%). Interest in future learning was high (UG: 82.5%; PG: 92.2%). Level of education was a significant predictor of knowledge (p<0.01), while male students showed more positive attitudes (p<0.01). First-year postgraduates reported better AI-related practices than other groups (p<0.01).

**Conclusion:**

Although most dental students lacked sufficient knowledge regarding the use of AI in dentistry, they displayed positive and encouraging attitudes toward its application. A large proportion expressed willingness to learn AI technologies to apply them in clinical practice. It is therefore recommended that universities and government bodies work together to integrate AI related topics into the dental curriculum to strengthen dental education in India.

## Introduction

The Fourth Industrial Revolution has reshaped how humans live, work, and interact, with technological advances rapidly influencing multiple sectors. ^1^ Among these, Artificial Intelligence (AI) and Augmented Intelligence (AUI) have drawn significant attention and are now increasingly integrated into healthcare and higher education. ^2^ AI can be broadly described as a computer-based simulation of human cognitive abilities, capable of performing tasks such as speech recognition, natural language comprehension, and complex decision-making. ^3^ The term *artificial intelligence* was first introduced by John McCarthy at Dartmouth University in 1956, and since then, AI has been widely regarded as a transformative force across diverse domains, including supply chain management, transportation, and healthcare. ^4^

The rapid rise of AI applications in the last decade can be attributed to breakthroughs in advanced algorithms, cost-efficient computing resources such as Graphic Processing Units (GPUs), and the availability of extensive annotated datasets. ^5^ Dentistry, in particular, has recently witnessed significant advances through AI-driven technologies. Following the basic computational framework of input, processing, and output, AI systems can process diverse data types in dentistry ranging from auditory data (e.g., handpiece sounds) and textual information (e.g., patient records, treatment parameters) to image-based data (e.g., radiographs and clinical photographs). ^6^ These inputs, when processed through neural networks, yield outputs such as diagnostic insights, prognoses, treatment planning, or disease prediction. ^7^ AI models, including conventional neural networks and advanced deep learning approaches, have already been applied in root canal anatomy analysis, staging of malignant lesions, detection of proximal caries, Computer Aided Design and Computer Aided Manufacturing (CAD/CAM)-based prosthesis design, and dental implant placement. ^8^

AI has become an essential component of dental healthcare education. In both preclinical and clinical settings, AI tools enhance learning experiences by offering adaptive, personalized, and mobile-based education. ^9^ AI systems can tailor learning resources to individual student needs, address knowledge gaps, and provide real-time feedback. ^10^ Furthermore, AI facilitates the continuous monitoring of academic progress, clinical exposure, and professional development while supporting mentorship and career guidance. These features have positioned AI as an invaluable resource in competency-based education, which is becoming increasingly relevant in global dental curricula. ^11^

Despite its potential, AI integration remains uneven across regions. While Western countries such as United States have incorporated AI into healthcare education and practice for decision support, ^12^ developing nations face infrastructural, financial, and sociocultural barriers. For example, India’s healthcare sector struggles with challenges such as limited institutional investment, resistance from healthcare providers, inadequate numbers of AI-trained professionals, and ethical concerns surrounding patient data security. ^13, 14^ Additionally, issues such as medicolegal implications, public perception, and fears of physician replacement further hinder the acceptance of AI in medical and dental practice. Addressing these challenges will require targeted investments, robust data policies, faculty training programs, and collaborative research across global institutions. ^15^

Although AI applications in dentistry are expanding, there remains a paucity of research exploring the knowledge, attitudes, and practices (KAP) of dental students regarding AI in India. Understanding how undergraduate and postgraduate dental students perceive and engage with AI is crucial for developing effective curricula, improving clinical training, and fostering responsible adoption of these technologies. This research aims to assess the level of awareness, attitudes, and readiness for AI integration among dental students, thereby contributing evidence to guide educational policies and infrastructure development in India and other developing countries.

## Methods

### Study design

This descriptive cross-sectional study was conducted between January and March 2024 and is reported in accordance with the Strengthening the Reporting of Observational Studies in Epidemiology (STROBE) guidelines.

### Study participants

The study included all the undergraduate dental students (final-year BDS students and house surgeons) and postgraduate dental students (MDS) from Bapuji Dental College and Hospital. Students who provided informed consent and completed the full questionnaire were eligible for analysis.

### Survey instrument

The questionnaire was adapted from a validated survey originally developed for medical students in Syria ^16^ and modified for dental students in India. The modified questions consisted of 21 close-ended items divided into two sections: The questionnaire consisted of two main sections. The first section had three questions related to demographic details of the participants. The second main section had 18 questions related to Knowledge, Attitude, and Practice (KAP) related to Artificial Intelligence (AI) in dentistry, subdivided into:

*Knowledge subscale* – Six items assessed awareness of AI, its subtypes, and applications in dentistry (oral radiology, oral surgery, and postgraduate training). Responses were scored as Yes = 1, No = 0. A total score >3 indicated *good knowledge.*

*Attitude subscale:* Seven items assessed perceptions of AI’s importance in dentistry, curriculum integration, diagnostic support, role in specialties, potential to replace dentists, and burden on clinicians. Responses were rated on a 5-point Likert scale (Strongly Agree = 5 to Strongly Disagree = 1). A score >5 indicated a *positive attitude.*

*Practice subscale:* Five assessed AI use in academics/clinical practice, ease of application, clinician’s role, and willingness to learn AI. Responses were Yes = 1, No/Never applied = 0. A score >2 indicated *good practice.*

The questionnaire was pilot tested on 20 students for validity and reliability. The test re-test score ranged from 0.8 to 0.9 (Knowledge = 0.8, Attitude = 0.82 and practice = 0.9) and the Cronbach’s alpha score ranged from 0.78 to 0.9 (Knowledge = 0.78, Attitude = 0.84 and practice = 0.9) indicating acceptable internal consistency.

### Administration of the survey

The questionnaire was distributed electronically via Google Forms to the official WhatsApp numbers registered with the college administration. The form included the survey title, purpose, consent statement, questionnaire items, and a thank-you note. Participants were given two days to respond. Non-respondents received up to two reminders at one-week intervals. Students who did not respond after the reminders were excluded from the study.

### Statistical analysis

Data were analyzed using IBM SPSS Statistics version 25.0 (IBM Corp., Armonk, NY, USA). Descriptive statistics (mean, standard deviation, frequencies, and percentages) summarized responses. Pearson’s chi-square test and Fisher’s exact test compared categorical variables. Mann–Whitney U was used to compared non-parametric data. A binary logistic regression was used to identify demographic predictors of KAP scores. Results were reported as unadjusted odds ratios (ORs) with 95% confidence intervals (CIs). A p-value <0.05 was considered statistically significant.

## Results

Out of 254 dental students invited to participate, 208 completed the survey, resulting in a response rate of 81.8%. The average age of participants was 24.29 ± 2.48 years, ranging from 21 to 45 years. The group consisted of 146 females (70.2%) and 62 males (29.8%). Among them, 120 were undergraduate (UG) students and 88 were postgraduate (PG) students. Specifically, the sample included 44 (36.5%) final-year BDS students, 76 (21.2%) house surgeons, 34 (16.3%) first-year MDS students, 30 (14.4%) second-year MDS students, and 24 (11.5%) third-year MDS students [ Table 1].

**Table 1.** Demographic characteristics of study participants.

Variables	N	Percentages	Mean ± SD
*Age*	208		24.29 ± 2.48
*Gender*			
Female	146	70.2	
Male	62	29.8	
*Class*			
Final year BDS	44	21.2	
Interns	76	36.5	
1 ^st^ year MDS	34	16.3	
2 ^nd^ year MDS	30	14.4	
3 ^rd^ year MDS	24	11.5	

### Knowledge of AI

When asked about their basic understanding of artificial intelligence (AI), 114 UG students (95%) and 85 PG students (96.6%) reported familiarity with the term. Awareness of AI subfields, such as machine learning and deep learning, was much higher among PG students (73; 83%) compared to UG students (32; 26.7%) (p = 0.001). Similarly, 80 PG students (90.9%) were familiar with the applications of AI in dentistry, compared to only 31 UG students (25.8%) (p = 0.004). However, when asked about specific uses of AI within dentistry, 68 PG students (77.3%) identified applications in oral radiology, while 58 (65.9%) recognized its role in oral surgery (p = 0.002). A majority of PG respondents (71; 80.6%) indicated that AI is not currently included in their curriculum [ Table 2].

**Table 2.** Comparison of knowledge on artificial intelligence between undergraduate and post-graduate students.

Questions	Response	Undergraduate students	Postgraduate students	p-value
Do you know what artificial intelligence is?	Yes	114 (95%)	85 (96.6%)	0.577
	No	6 (5%)	3 (3.4%)	
Do you know about machine learning and deep learning (subtypes of AI)?	Yes	32 (26.7%)	73 (83%)	0.001
	No	88 (73.3%)	15 (17%)	
Do you know about any application of AI in the dental field?	Yes	31 (25.8%)	80 (90.9%)	0.004
	No	89 (74.2%)	8 (9.1%)	
Do you know about the application of AI in oral radiology field?	Yes	28 (23.4%)	68 (77.3%)	0.002
	No	92 (76.6%)	20 (22.7%)	
Do you know about the application of AI in the oral surgery field?	Yes	12 (10%)	58 (65.9%)	0.001
	No	108 (90%)	30 (34.1%)	
If you are a PG student, does your training include a curriculum regarding AI?	Yes	-	7 (19.4%)	
	No	-	71 (80.6%)	

The overall mean knowledge score was 4.32 ± 1.79. No significant difference was found between genders (p = 0.177). PG students scored significantly higher (5.12 ± 1.67) than UG students (3.66 ± 1.60) (p = 0.000) [ Table 5]. Regardless of age and gender, AI knowledge levels were generally good. Final-year MDS students demonstrated particularly strong knowledge (p = 0.003) [ Table 6]. The regression analysis confirmed that the level of dental education is a significant predictor of AI knowledge, with final-year MDS students showing the highest level of understanding compared to other groups [ Table 7].

**Table 3.** Comparison of attitude on artificial intelligence between undergraduate and post-graduate students.

Questions	Response	Undergraduate students	Postgraduate students	p-value
Do you believe AI is essential in the dental field?	Strongly agree	21 (17.5%)	37 (42%)	0.001
	Agree	76 (63.3%)	37 (42%)	
	Neutral	23 (19.2%)	12 (13.6%)	
	Disagree	0	1 (1.1%)	
	Strongly disagree	0	1 (1.1%)	
Do you think AI should be included in the curriculum in dental school as well as specialist training?	Strongly agree	22 (18.3%)	28 (31.8%)	0.05
	Agree	74 (61.7%)	47 (53.4%)	
	Neutral	19 (15.8%)	10 (11.4%)	
	Disagree	5 (4.2%)	1 (1.1%)	
	Strongly disagree	0	2 (2.3%)	
Do you think that AI aids practitioners in early diagnosis and assessment of the severity of disease?	Strongly agree	22 (18.3%)	19 (21.6%)	0.525
	Agree	72 (60%)	51 (58%)	
	Neutral	20 (16.7%)	17 (19.3%)	
	Disagree	3 (2.5%)	1 (1.1%)	
	Strongly disagree	3 (2.5%)	0	
Do you believe that AI will replace physicians in the future?	Strongly agree	11 (9.2%)	5 (5.7%)	0.184
	Agree	29 (24.2%)	33 (37.5%)	
	Neutral	36 (30%)	18 (20.5%)	
	Disagree	35 (29.2%)	23 (26.1%)	
	Strongly disagree	9 (7.5%)	9 (10.2%)	
Do you believe AI is very essential in the field of radiology?	Strongly agree	22 (18.3%)	19 (21.6%)	0.271
	Agree	73 (60.8%)	45 (51.1%)	
	Neutral	19 (15.8%)	21 (23.9%)	
	Disagree	3 (2.5%)	3 (3.4%)	
	Strongly disagree	3 (2.5%)	0	
Do You believe AI is essential in the field of oral surgery?	Strongly agree	16 (13.3%)	14 (15.9%)	0.866
	Agree	71 (59.2%)	49 (55.7%)	
	Neutral	26 (21.7%)	22 (25%)	
	Disagree	4 (3.3%)	2 (2.3%)	
	Strongly disagree	3 (2.5%)	1 (1.1%)	
Do you believe AI would be a burden for practitioners?	Strongly agree	10 (8.3%)	7 (8%)	0.081
	Agree	27 (22.5%)	31 (35.2%)	
	Neutral	40 (33.3%)	18 (20.5%)	
	Disagree	39 (32.5%)	25 (28.4%)	
	Strongly disagree	4 (3.3%)	7 (8%)	

**Table 4.** Comparison of practice of artificial intelligence between undergraduate and post-graduate students.

Questions	Response	Undergraduate students	Postgraduate students	p-value
Have you ever applied AI technology in any field?	Yes	8 (6.7%)	54 (61.4%)	0.04
	No	112 (93.3%)	34 (36.6%)	
Was it easy for you to apply AI?	Yes	4 (3.3%)	18 (20.4%)	0.50
	No	4 (3.3%)	36 (40.9%)	
	Never applied	112 (93.3%)	34 (36.6%)	
Did AI make your task easy?	Yes	7 (87.5%)	50 (92.6%)	0.014
	No	1 (12.5%)	4 (7.4%)	
Clinician role is important in application and evaluation of AI in the dental field	Yes	69 (57.5%)	65 (73.9%)	0.047
	No	16 (13.3%)	6 (6.8%)	
	Don’t know	35 (29.2%)	17 (19.3%)	
Would you like to work on AI in future?	Yes	86 (71.7%)	74 (84.1%)	0.060
	No	13 (10.8%)	8 (9.1%)	
	Don’t know	21 (17.5%)	6 (6.8%)	

**Table 5.** Mean knowledge, attitude and practice scores and demographic details of study participants.

Variables	N	Demographic details	Mean ± SD	p-value
Mean knowledge of AI	62	Male	4.58 ± 1.82	0.177
	146	Female	4.21 ± 1.78	
	120	Undergraduate students	3.66 ± 1.60	0.000
	88	Postgraduate students	5.12 ± 1.67	
Mean attitude of AI	62	Male	5.16 ± 1.73	0.014
	146	Female	4.43 ± 2.00	
	120	Undergraduate students	4.55 ± 1.88	0.372
	88	Postgraduate students	4.79 ± 2.05	
Mean practice of AI	62	Male	3.51 ± 1.66	0.087
	146	Female	3.06 ± 1.73	
	120	Undergraduate students	2.88 ± 1.78	0.002
	88	Postgraduate students	3.63 ± 1.54	

**Table 6.** Knowledge, attitude and practice scores and demographic details of study participants.

Demographic details	Variables		p-value
	**Knowledge of AI**		
*Age group*	**Good N(%)**	**Poor N(%)**	0.269
21-25 years	92 (61.7%)	57 (38.3%)	
26-30 years	42 (72.4%)	16 (27.6%)	
>31 years	1 (100%)	0	
*Gender*			0.809
Male	41 (66.1%)	21 (33.9%)	
Female	94 (64.4%)	52 (35.6%)	
*Course*			0.003
Final year BDS	24 (54.5%)	20 (45.5%)	
Internship	41 (53.9%)	35 (46.1%)	
1 ^st^ MDS	28 (82.4%)	6 (17.6%)	
2 ^nd^ MDS	21 (70%)	9 (30%)	
3 ^rd^ MDS	21 (87.5%)	3 (12.5%)	
	**Attitude of AI**		
*Age group*	**Positive**	**Negative**	0.733
21-25 years	101 (67.8%)	48 (32.2%)	
26-30 years	41 (70.7%)	17 (29.3%)	
>31 years	1 (100%)	0	
*Gender*			0.037
Male	49 (79%)	13 (21%)	
Female	94 (64.4%)	52 (35.6%)	
*Course*			0.641
Final year BDS	28 (63.6%)	16 (36.4%)	
Internship	52 (68.4%)	24 (31.6%)	
1 ^st^ MDS	25 (73.5%)	9 (26.5%)	
2 ^nd^ MDS	19 (63.3%)	11 (36.7%)	
3 ^rd^ MDS	19 (79.2%)	5 (20.8%)	
	**Practice of AI**		
*Age group*	**Good**	**Poor**	0.516
21-25 years	71 (47.7%)	78 (52.3%)	
26-30 years	30 (51.7%)	28 (48.3%)	
>31 years	1 (100%)	0	
*Gender*			0.163
Male	35 (56.5%)	27 (43.5%)	
Female	67 (45.9%)	79 (54.1%)	
*Course*			0.001
Final year BDS	9 (20.5%)	35 (79.5%)	
Internship	40 (52.6%)	36 (47.4%)	
1 ^st^ MDS	21 (61.8%)	13 (38.2%)	
2 ^nd^ MDS	18 (60%)	12 (40%)	
3 ^rd^ MDS	14 (58.3%)	10 (41.7%)	

**Table 7.** Binary logistic regression between knowledge, attitude, practice and demographic details of study participants.

Demographic details	Categories	Odds ratio	p-value	Lower	Upper
	**Knowledge of AI**				
Gender	Female	Reference			
	Male	0.889	0.729	0.459	1.722
Class	Final year BDS	Reference			
	Internship	0.154	0.001	0.010	0.296
	1 ^st^ MDS	0.152	0.001	0.009	0.308
	2 ^nd^ MDS	0.471	0.338	0.101	2.194
	3 ^rd^ MDS	0.798	0.038	0.043	0.197
	**Attitude of AI**				
Gender	Female	Reference			
	Male	0.461	0.032	0.228	0.935
Class	Final year BDS	Reference			
	Internship	0.570	0.320	0.188	1.725
	1 ^st^ MDS	0.444	0.175	0.137	1.435
	2 ^nd^ MDS	0.741	0.640	0.211	2.602
	3 ^rd^ MDS	0.422	0.176	0.121	1.473
	**Practice of AI**				
Gender	Female	Reference			
	Male	0.606	0.123	0.321	1.145
Class	Final year BDS	Reference			
	Internship	0.797	0.635	0.313	2.029
	1 ^st^ MDS	0.177	0.002	0.059	0.534
	2 ^nd^ MDS	1.171	0.773	0.401	3.423
	3 ^rd^ MDS	1.033	0.953	0.344	3.103

### Attitude toward AI

Only a small proportion of students strongly agreed that AI is vital in dentistry 21 undergraduates (17.5%) and 37 postgraduates (42%) (p = 0.001). About 33.4% of UG students and 43.2 % of PG students agreed that AI would replace dentists in future. Similarly, 22 UG students (18.3%) and 28 PG students (31.8%) strongly agreed that AI should be incorporated into both dental school curricula and specialist training. A majority of participants 72 UG (60%) and 51 PG (58%) students agreed that AI supports practitioners in the early detection of disease and in assessing its severity. Very few, 11 UG (9.2%) and 5 PG (5.7%) students, strongly agreed that AI would eventually replace physicians.

Most respondents recognized AI’s importance in specific specialties: 73 UG students (60.8%) and 45 PG students (51.1%) agreed that it is highly valuable in radiology, while 71 UG (59.2%) and 49 PG (55.7%) students agreed that it plays an essential role in oral surgery. Conversely, only 10 UG (8.3%) and 7 PG (8%) students strongly agreed that AI could be burdensome for practitioners [ Table 3].

The mean attitude score toward AI was 4.65 ± 1.95. A significant gender difference was observed, with male students showing a more favourable attitude (5.16 ± 1.73; p = 0.014). Regardless of age or academic level, the majority of participants expressed positive views about AI. Notably, 49 male respondents (79%) demonstrated a positive attitude, significantly higher compared to females (p = 0.037) [ Table 6]. Further analysis indicated that male gender is a significant predictor of positive attitudes toward AI [ Table 7].

### Practice of AI

More than half of the postgraduate (PG) respondents, 54 (61.4%), reported using AI technology compared to only 8 undergraduate (UG) students (6.7%) (p = 0.04). Among PG students, 36 (40.9%) reported difficulties in applying AI. Nevertheless, most of those who had used AI noted that it made tasks easier. A majority of PG students, 65 (73.9%), emphasized the critical role of clinicians in diagnosis and treatment, compared to 69 UG students (57.5%) (p = 0.047). Interest in learning more about AI was high among both groups, with 86 UG (71.7%) and 74 PG (84.1%) students expressing interest [ Table 4].

The overall mean practice score for AI was 3.20 ± 1.72. Male students had higher scores (3.51 ± 1.66) than females (3.06 ± 1.73), although the difference was not statistically significant (p = 0.087). PG students, however, achieved significantly higher practice scores (3.63 ± 1.54) compared to UG students (2.88 ± 1.78) (p = 0.002) [ Table 5]. Among participants aged 21–25 years, 78 (52.3%) had low practice scores, but the difference across age groups was not significant (p = 0.516). While 35 male students (56.5%) demonstrated good practice scores compared to female students, the difference remained statistically insignificant (p = 0.163). Importantly, PG students particularly those in the first year of MDS were identified as having significantly better practice of AI compared to other groups (p = 0.001) [ Table 6, Table 7].

## Discussion

The key findings of this study indicate that most students were familiar with the term AI, postgraduate students had greater awareness of AI applications in medical and dental fields, a considerable proportion of postgraduate students found AI easier to apply compared to undergraduates, and the majority of respondents emphasized the indispensable role of clinicians in AI-assisted dentistry. These outcomes suggest that dental students remain in an exploratory phase, with a degree of uncertainty about adopting AI applications in their field.

The gender distribution of participants in this survey (70.2% female, 29.8% male) aligns with previous reports by Murali et al. ^17^ (73% female, 27% male) and Elhijazi et al. ^18^ (71.4% female, 28.9% male). The highest participation was from interns (36.5%), while final-year MDS students were least represented (11.5%). By contrast, Murali et al. ^17^ reported lower participation from interns (19.3%) and higher involvement of postgraduates (20.7%), whereas Elchaghaby et al. found most respondents were fifth-year students (53%) and fewest were in their third year (21%). ^19^

In the present study, 95.6% of participants had basic knowledge of AI, closely matching Murali et al.’s findings (94%). Awareness of machine learning and deep learning was higher among PG students (82.9%), likely due to the integration of AI into clinical practice and increased exploration of new technologies through social media and smartphones. Supporting this, Yüzbaşıoğlu ^20^ reported that 76.1% of students learned about AI from social media, while Aldowah et al. ^21^ found similar results (78%). Compared with medical students in Syria ^16^ (34.7%) and Pakistan ^22^ (35.3%), our respondents demonstrated higher knowledge of AI subtypes. However, only 25.8% of UG students knew about AI’s applications in dentistry, versus 90.9% of PG students who were aware of its role in healthcare. This disparity reflects the limited academic and clinical exposure of undergraduates to AI, underscoring the need for curriculum integration. Medical studies from Syria ^16^ (87.4%), Pakistan ^20^ (74.4%), and the UK ^23^ (78%) further reinforce AI’s perceived importance in healthcare. Similarly, 77.3% of our PG students acknowledged its role in oral radiology, consistent with reports from Pakistan ^22^ (74%) and Syria ^16^ (73%).

Regarding future implications, 33.4% of UG and 43.2% of PG students believed AI might replace dentists. These findings contrast with Jeong’s study ^24^ where 72% disagreed with this notion, but align with Elhijazi ^18^ where 53.7% considered replacement possible. The higher proportion in our study may stem from misconceptions about AI’s scope and limitations in dentistry. Nonetheless, most participants (77.8%) agreed that AI supports early diagnosis and disease severity assessment, echoing global trends. AI systems have already demonstrated their value in enhancing diagnostic accuracy and reducing human error. Additionally, more than half of respondents (57.7%) acknowledged AI’s importance in oral surgery, a view shared by students in Turkey, ^20^ Saudi Arabia, ^25^ and India. ^26^

Interestingly, 36% of students felt AI could become a burden for practitioners. While AI is revolutionizing decision-making and personalized treatment, its integration also raises concerns regarding job displacement, information overload, and potential erosion of clinical skills if overreliance occurs. To prevent disruption, AI should be positioned as a supportive tool rather than a substitute in oral healthcare.

Overall, the findings show moderate baseline knowledge of AI, broad acceptance of its inclusion in the curriculum, and a generally positive attitude toward its use in dentistry. Students also demonstrated enthusiasm for future learning. Therefore, we recommend hands on workshops, seminars, and training programs on machine learning and deep learning in clinical dentistry. This would enable future dentists to harness AI effectively, reducing errors and enhancing treatment quality. Addressing identified knowledge gaps will require expanding AI training within dental education to eliminate misconceptions and prepare these students for the future of digital dentistry.

### Limitations

This study has several limitations. First, its cross-sectional design captures responses at a single point in time and therefore does not allow causal inferences. Second, data were collected from a single institution, which may limit the generalizability of the findings to other regions of India. Nonetheless, internal validity was strengthened through the use of a validated questionnaire, a high response rate, and inclusion of both undergraduate and postgraduate students across all academic levels. Future research should include multi-institutional studies with larger and more diverse samples. Longitudinal designs are also recommended to assess how exposure to AI training influences the development of students’ knowledge, attitudes, and competencies over time.

## Conclusion

A significant proportion of dental students in this study demonstrated basic knowledge of AI and a positive attitude toward incorporating it into the dental curriculum. Students recognized its value in dental applications and expressed interest in future learning. Universities and policymakers should collaborate to integrate AI training into dental education, equipping future dentists to utilize AI in routine practice and ultimately improve the quality of dental healthcare.

## Ethics and consent

The protocol was approved by the Institutional Review Board of Bapuji Dental College and Hospital, India (Approval number: ECR/1652/Inst/KA/2022/24-04/08-007). The Committee on Research Ethics followed internationally recognized guidelines for the protection of human subjects, including the Declaration of Helsinki, the Belmont Report, and CIOMS principles. Prior to participation, all individuals were provided with a clear explanation of the study and gave informed consent electronically. The consent form described the study’s purpose, estimated completion time, and was written in simple, accessible language. Participation was entirely voluntary, and responses were collected anonymously. Participants were informed of their right to withdraw at any point, and assured that their personal information would remain confidential. Access to the dataset was restricted to the principal investigator.

## Data Availability

Figshare – **Knowledge, Attitudes, and Practices of Artificial Intelligence in Dentistry: a cross-sectional survey**. https://doi.org/10.6084/m9.figshare.30513776.v1. ^27^ This project contains following underlying data:
• study-data.xlsx Data are available under the terms of the Creative Commons Zero “No rights reserved” data waiver (CC BY 4.0 Public domain dedication). Figshare - **Knowledge, Attitudes, and Practices of Artificial Intelligence in Dentistry: a cross-sectional survey** (Questionnaire), https://doi.org/10.6084/m9.figshare.30513803.v1. ^28^ This project contains following extended data
• Survey questionnaire, participant information and consent form.docx Data are available under the terms of the Creative Commons Zero “No rights reserved” data waiver (CC BY 4.0 Public domain dedication).
