# Supplementary material for: Knowledge, Attitudes, and Practices of Artificial Intelligence in Dentistry: A cross-sectional survey
Source: F1000Res. 2026 Jan 28;14:1314. Originally published 2025 Nov 26. [Version 2] doi: 10.12688/f1000research.173028.2 (PMC12780518; doi:10.12688/f1000research.173028.2)
Supplement: Peer review history

# Reviewer report: Reviewer response for version 2

**DOI:** 10.5256/f1000research.195508.r453481

**Published:** 2026-02-06

**Authors:** Dr Praveen Gangadharappa

**Affiliations:** 1 Jazan University, Jazan, Saudi Arabia

no changes required

Is the work clearly and accurately presented and does it cite the current literature?

Yes

If applicable, is the statistical analysis and its interpretation appropriate?

I cannot comment. A qualified statistician is required.

Are all the source data underlying the results available to ensure full reproducibility?

No source data required

Is the study design appropriate and is the work technically sound?

Yes

Are the conclusions drawn adequately supported by the results?

Yes

Are sufficient details of methods and analysis provided to allow replication by others?

Yes

Reviewer Expertise:

clinical research,

I confirm that I have read this submission and believe that I have an appropriate level of expertise to confirm that it is of an acceptable scientific standard.

# Reviewer report: Reviewer response for version 1

**DOI:** 10.5256/f1000research.190802.r436907

**Published:** 2026-01-07

**Authors:** Dr Praveen Gangadharappa

**Affiliations:** 1 Jazan University, Jazan, Saudi Arabia

**1. Incorrect interpretation of logistic regression (Table 7)**

 In **Table 7**, odds ratios (ORs) < 1 indicate **lower odds compared to reference**, but the manuscript interprets some of these incorrectly.

**Examples:**

- **Knowledge of AI**
  - Internship OR = **0.154**, p = 0.001
  - 1st MDS OR = **0.152**, p = 0.001

Yet the text states:

 “Final-year MDS students demonstrated particularly strong knowledge…”

**2. Mismatch between mean scores and categorical KAP classification**

 You use:

- Mean scores (Table 5)
- Dichotomized “Good / Poor” KAP scores (Tables 6 & 7)

But **cut-offs are not statistically justified**.

 Example:

- Knowledge score range = 0–6
- “Good knowledge” defined as >3

Yet:

- Mean knowledge = 4.32 ± 1.79
- Still, **~35% are classified as poor**

**3. Overstatement in conclusion**

 The conclusion states:

 “A significant proportion of dental students demonstrated basic knowledge of AI…”

 But:

- UG students had **low application awareness (25.8%)**
- Practice scores were **poor in more than half**

**4. Single-institution sample limits generalizability**

 You mention this in limitations, but the **Abstract and Conclusion still generalize to “dental students in India.”**

 5. **Practice results wording**

 Statement:

 “A considerable proportion of undergraduate students found AI easier to apply compared to postgraduates”

 But:

- Only **4 UG students** reported ease
- Most UG **never applied AI**

6. **Terminology consistency**

- “Physicians” is used instead of **dentists** in some attitude questions and discussion.
- This creates conceptual ambiguity.

Is the work clearly and accurately presented and does it cite the current literature?

Yes

If applicable, is the statistical analysis and its interpretation appropriate?

I cannot comment. A qualified statistician is required.

Are all the source data underlying the results available to ensure full reproducibility?

No source data required

Is the study design appropriate and is the work technically sound?

Yes

Are the conclusions drawn adequately supported by the results?

Yes

Are sufficient details of methods and analysis provided to allow replication by others?

Yes

Reviewer Expertise:

clinical research,

I confirm that I have read this submission and believe that I have an appropriate level of expertise to confirm that it is of an acceptable scientific standard.

# Reviewer report: Reviewer response for version 1

**DOI:** 10.5256/f1000research.190802.r436900

**Published:** 2025-12-11

**Authors:** Pankaj Kukreja

**Affiliations:** 1 Faculty of dentistry, Department of Biomedical Dental Sciences, Al Baha University (Ringgold ID: 158203), Al Bahah, Al Bahah Province, Saudi Arabia

The article is well written. Appropriate methodology has been followed. The article effectively spans radiology and pathology domains of dentistry related to understanding of both postgraduate and undergraduate students and ensures that readers gain an understanding of AI’s impact on the students. The authors not only summarize existing studies but also highlight limitations which adds depth and balance to the discussion section. The discussion provides valuable guidance for future research. While the article is already strong, future iterations could explore patient perspectives on AI integration in dentistry to enrich the human-centered dimension.

Is the work clearly and accurately presented and does it cite the current literature?

Yes

If applicable, is the statistical analysis and its interpretation appropriate?

Yes

Are all the source data underlying the results available to ensure full reproducibility?

Yes

Is the study design appropriate and is the work technically sound?

Yes

Are the conclusions drawn adequately supported by the results?

Yes

Are sufficient details of methods and analysis provided to allow replication by others?

Yes

Reviewer Expertise:

Oral and Maxillofacial surgery, Dental implantology

I confirm that I have read this submission and believe that I have an appropriate level of expertise to confirm that it is of an acceptable scientific standard.

# Reviewer report: Reviewer response for version 1

**DOI:** 10.5256/f1000research.190802.r436903

**Published:** 2025-12-11

**Authors:** Laliytha Kumar Bijai

**Affiliations:** 1 King Saud bin Abdulaziz University for Health Sciences, Riyadh, Riyadh Province, Saudi Arabia

The manuscript is well written. 

 However, it would be more plausible to readers if the following were addressed.

 1. One part of the result does not coincide with the first line of the discussion. 

 In the results section,  only 6.7% of UGs had ever used AI (vs. 61.4% of PGs).

 Can you justify why, in the discussion section, it is mentioned that the study reports that UGs found AI “easier to apply” than PGs?

 This could be either a misunderstanding of the question item or ambiguity in how “ease of application” was measured. 

 2. The limitation clearly states that the KAP study cannot be generalized as it was done only in one institution. So it is fair to modify the sentence of recommendation to Universities and policymakers. Instead, you can conclude that only if we have multi-institutional studies can we make a substantial recommendation to the policymakers.

Is the work clearly and accurately presented and does it cite the current literature?

Yes

If applicable, is the statistical analysis and its interpretation appropriate?

Yes

Are all the source data underlying the results available to ensure full reproducibility?

Yes

Is the study design appropriate and is the work technically sound?

Yes

Are the conclusions drawn adequately supported by the results?

Partly

Are sufficient details of methods and analysis provided to allow replication by others?

Yes

Reviewer Expertise:

Oral Cancer, Oral potentially malignant disorders, prediction of malignant transformation,

I confirm that I have read this submission and believe that I have an appropriate level of expertise to confirm that it is of an acceptable scientific standard, however I have significant reservations, as outlined above.
